# Toward the Prediction of FBPase Inhibitory Activity Using Chemoinformatic Methods

**DOI:** 10.3390/ijms13067015

**Published:** 2012-06-07

**Authors:** Ming Hao, Shuwei Zhang, Jieshan Qiu

**Affiliations:** Department of Materials Science and Chemical Engineering, Dalian University of Technology, Dalian 116023, Liaoning, China; E-Mails: dluthm@yeah.net (M.H.); zswei@dlut.edu.cn (S.Z.)

**Keywords:** FBPase inhibitor, chemoinformatics methods, genetic algorithm, random forest

## Abstract

Currently, Chemoinformatic methods are used to perform the prediction for FBPase inhibitory activity. A genetic algorithm-random forest coupled method (GA-RF) was proposed to predict fructose 1,6-bisphosphatase (FBPase) inhibitors to treat type 2 diabetes mellitus using the Mold^2^ molecular descriptors. A data set of 126 oxazole and thiazole analogs was used to derive the GA-RF model, yielding the significant non-cross-validated correlation coefficient *r*^2^_ncv_ and cross-validated *r*^2^_cv_ values of 0.96 and 0.67 for the training set, respectively. The statistically significant model was validated by a test set of 64 compounds, producing the prediction correlation coefficient *r*^2^_pred_ of 0.90. More importantly, the building GA-RF model also passed through various criteria suggested by Tropsha and Roy with *r*^2^_o_ and *r*^2^_m_ values of 0.90 and 0.83, respectively. In order to compare with the GA-RF model, a pure RF model developed based on the full descriptors was performed as well for the same data set. The resulting GA-RF model with significantly internal and external prediction capacities is beneficial to the prediction of potential oxazole and thiazole series of FBPase inhibitors prior to chemical synthesis in drug discovery programs.

## 1. Introduction

Diabetes is one of the most prevalent diseases worldwide, and the incidence of this continues to grow, which causes a global public health burden. It is estimated that by 2025, India, China and the United States will possess the largest number of people with diabetes [1]. The more prevalent form, type 2 diabetes, accounts for more than 90% of cases. The pathogenesis of type 2 diabetes is complex, involving progressive development of insulin resistance and a relative deficiency in insulin secretion, which may lead to overt hyperglycemia [2]. Type 2 diabetes is associated with the metabolic syndrome that comprises a set of alterations that include glucose intolerance, truncal obesity, hypertension and dyslipidemia [3]. It has been reported that the metabolic syndrome is associated with a markedly increased risk of coronary artery disease [4]. The risk of myocardial infarction in patients with diabetes and no history of cardiac disease roughly equates the risk in non-diabetic patients with known cardiac diseases [5]. In addition, diabetes often conspires to cause various complications such as eye diseases. It has been documented that diabetes is the primary reason for loss of vision [6]. Patients with diabetes suffer from great mental and physical pain. Consequently, it is necessary to develop an effective drug for the treatment of this disease. Since hyperglycemia leads to severe microvascular and macrovascular complications, the primary treatment goal is to reduce the glucose level. Several popular classes of oral diabetes therapies on the market include sulfonylureas, peroxisome proliferator-activated receptor-γ (PPAR-γ) agonists, metformin and so forth [7,8], which lower glucose by increasing glucose metabolism either via enhanced insulin secretion or improved insulin sensitivity. However, therapies with these drugs still have several drawbacks. For example, sulfonylurea therapy is usually associated with weight gain [9] and metformin therapy is forbidden in patients with renal and hepatic diseases, respiratory insufficiency and alcohol abuse [10]. As a consequence, there is a need for novel, more effective drugs with more safe profiles and fewer side effects to the treatment of diabetes.

Fructose 1,6-bisphosphatase (FBPase), a highly regulated enzyme that catalyzes the second to last step in gluconeogenesis, draws attention as a potential therapeutic target to treat type 2 diabetes mellitus [10,11]. FBPase enables the inhibition of gluconeogenesis from all the corresponding substrates while avoiding direct effects on glycogenolysis, glycolysis and the tricarboxylic acid cycle. In addition, evidence from clinical research suggests that FBPase inhibitors may show an adequate safety margin [11]. Several classes of agents against FBPase have recently been reported including anilinoquinazolines [12], benzoxazole benzenesulfonamides [13], MDL-29951 [14], adenosine 5′-monophosphate (AMP) mimics, *etc*. Some of these series of drugs, however, were explored without successfully achieving acceptable oral bioavailability, thus, it is still required to research novel drugs with desirable characteristics.

Chemoinformatic methods, as a complementary approach of experiment technology, have found wide utility and acceptance, and these methods are playing a central role in drug design [15]. In view of this, previous reports [16] have performed a computational study based on a series of FBPase inhibitors. However, these methods were developed and tested on just a few compounds. In the current work, a larger dataset was used to derive a statistical model with a high prediction power.

Random forest (RF), a new regression tool, has been reported to be a combination of relatively high prediction accuracy and a collection of desired features that makes RF uniquely suited for modeling in chemoinformatics [17] based on a quantitative description of the compound’s molecular structure. RF can show an excellent performance even when most predictive variables are noise, it can be used when the number of variables is much larger than the number of observations, and it returns measures of variable importance. It is well known that an ideal regression model should have a high performance with few descriptors. Thus in the present work, to optimize the Mold^2^ molecular descriptor subset [18], with the statistical performance and efficiency of the model being simultaneously enhanced, the genetic algorithm-random forest coupled method is selected to perform a regression task to investigate whether the proposed GA-RF method can construct an ideal prediction model for the FBPase inhibitors. In addition, the derived optimal model was checked by Y-randomization to ensure that the prediction model was not obtained by chance correlation. Moreover, the rigorous statistical criteria suggested by Thropsha and Roy [19,20] were used to validate the model as well. For comparison with the GA-RF, the pure RF model, which means that the model was developed in the full descriptors without variable selection, was also applied using the same dataset.

## 2. Results and Discussion

### 2.1. Descriptor Calculation and Preprocessing

For the quantitative structure-activity relationship (QSAR) investigations, one of the important factors affecting the quality of the model is the choice of molecular descriptors used to obtain the structural information suitable for model development. The software Mold^2^ [18] enables a rapid calculation of a large and diverse set of descriptors encoding two-dimensional chemical structure information. A comparative analysis of Mold^2^ descriptors with those calculated by commercial tools such as Cerius^2^ [21], Dragon [22] on several data sets demonstrated that Mold^2^ descriptors can convey a similar amount of information as those widely-used software packages [18]. Although acting as a freely available tool, Mold^2^ has been proved suitable not only for the QSAR research [23–25], but also for virtual screening of large databases in drug development [18]. In the present work, a total of 777 Mold^2^ descriptors were calculated based on the SDF file format of all the studied FBPase inhibitors. All these descriptors were then preprocessed as follows: (1) descriptors containing values greater than 85% zero were removed; (2) zero- and near zero-variance predictors were removed, as descriptors like this may cause the model to crash or the fit to be unstable; and (3) one of the two descriptors that had absolute correlations above 0.75 was omitted. After these steps, the number of the descriptors was reduced to 108 for further research.

### 2.2. Split of the Training and Test Sets

**Figure 1:**
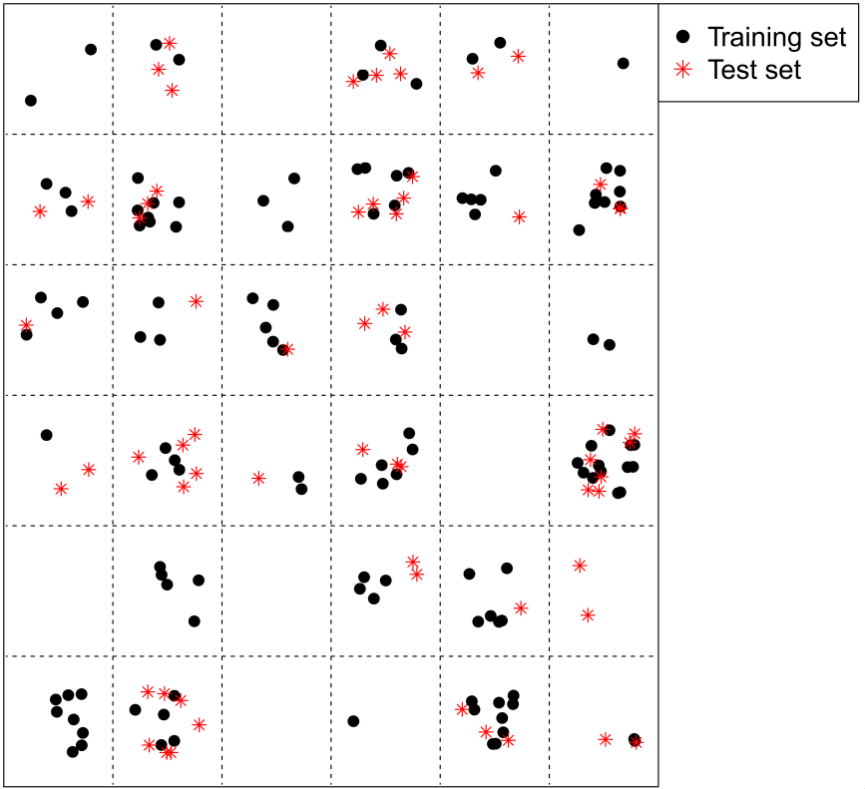
Self-organizing map (SOM) analysis for fructose 1,6-bisphosphatase (FBPase) inhibitors, where the black dot denotes the training set and the red asterisk stands for the test set.

Rational selection of training and test sets is also one of the important and challenging steps for the development of validated QSAR models. Self-organizing maps (SOM) can be employed for data survey, which has been successfully applied to split datasets [26,27]. In the current study, in order to probe the descriptor space, a total of 108 Mold^2^ descriptors were used to obtain a SOM map. A small Kohonen network with 6 × 6 = 36 neurons was employed, producing a map with 36 positions. All the compounds were placed onto the 36 positions of the Kohonen map. Figure 1 demonstrates the distribution of the compounds, where the black dot denotes the training set, while the red asterisk stands for the compounds from the test set. As can be seen from this figure, firstly, the representative points of the test set are close to those of the training set, and secondly, the training and test sets uniformly fill the whole chemical space, indicating a rational selection of the training and test inhibitors in the present work [28]. The training set was applied for the development of the model and the external test set was used for the assessment of the built model. The training set and test sets include 126 and 64 compounds, respectively.

### 2.3. Set Parameters of GA-RF Algorithm

In the present work, all the genetic parameters are set as follows: The number of maximum generations is set to 200 and the number of individuals to 50. Individuals are then selected from the population using the stochastic universal sampling algorithm, with a generation gap of 0.9. The double-point crossover is adopted with the probabilities of 0.7. The mutation operation is performed based on the default value included in the genetic algorithm toolbox developed by the Evolutionary Computation Research Team at The University of Sheffield, UK. Herein, the minimum out-of-bag (OOB) mean squared error (MSE) is used as the fitness function to obtain the optimal individual.

### 2.4. Statistical Results

Apart from the quality of the used data sets, the selection of proper descriptors relevant to the FBPase inhibitory activity is crucial for optimizing the prediction system by reducing the noise in a statistical learning process. After GA-RF, the final 40 Mold^2^ descriptors are selected. Table 1 illustrates the names of these selected descriptors and the corresponding definitions [18].

**Figure 2:**
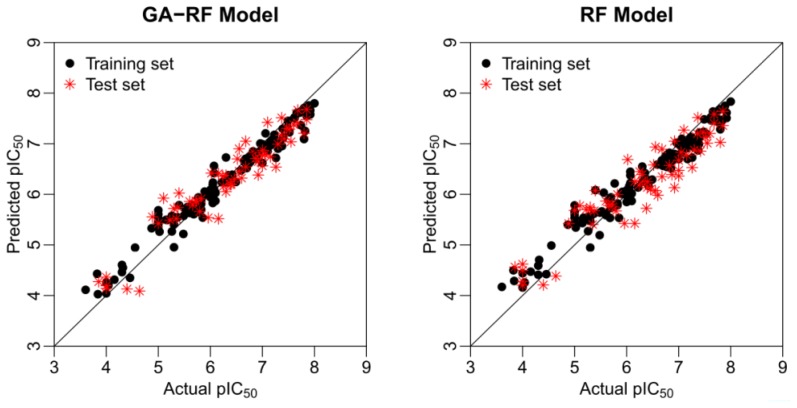
The scatter plots of actual and predicted activity by GA-RF and RF models.

Based on the determined optimal parameters by GA, the GA-RF model presents an (root-mean-square error) RMSE of 0.25 and 0.34 for the training and test sets, respectively. The determined coefficient *r*^2^_ncv_ reaches a value as high as 0.96 with *r*^2^_cv_ = 0.67 for the training set. The model predictability is evaluated by an external prediction set, which illustrates *r*^2^_ts_ and *r*^2^_pred_ values of 0.91 and 0.90, respectively. It is well known that the random forest algorithm can manipulate the data set even with a large number of descriptors [17]. Thus we compare the GA-RF with pure RF, which means that the latter model is built based on the whole 108 descriptors. It can be seen from Table 2 that the pure RF performs comparably but relatively low statistics compared with GA-RF. The scatter plots of the experimental *versus* predicted FBPase inhibitory activity based on the GA-RF and RF models are shown in Figure 2, where the proposed GA-RF model presents a relatively better performance than RF. For the former, the data points of training and test sets distribute more closely in a straight line (*y* = *x*), indicating that GA-RF exhibits both the inner and external prediction power. Table 3 gives the experimental and predicted results for both models.

### 2.5. Further Test for the External Prediction Power

To validate firmly the performances of the prediction, the squared correlation coefficient values between the observed and predicted values for the test set compounds with intercept (*r*^2^_ts_) and without intercept (*r*^2^_o_) are also calculated. Table 4 presents the values of the parameters for all models in the present work. According to references [19,34], models are considered acceptable if they satisfy all the following conditions: (1) *r*^2^_pred_ > 0.5, (2) *r*^2^_ts_ > 0.6, and (3) *r*^2^_o_ is close to *r*^2^_ts_, such that the [(*r*^2^_ts_ − *r*^2^_o_)/*r*^2^_ts_] < 0.1 and 0.85 ≤ k ≤ 1.15. When the predicted values of the test set compounds (*x* axis) are plotted against the observed values of the compounds (*y* axis) with the intercept set to zero, the slope of the fitted line gives the value of k, with the corresponding correlation coefficient *r*^2^_o_. Herein, *r*^2^_o_ is a quantity characterizing linear regression with the *y*-intercept set to zero, which can be illustrated by *y* = k*x*; while *r*^2^_ts_ is the conventional coefficient of determination for the best fit linear regression (*i.e*., denoted by *y* = a*x* + b) in the test set [19,28]. It can be noticed that the developed GA-RF and pure RF models fully satisfy all the requirements, but the latter is relatively less accurate than GA-RF.

Roy *et al*. have reported [35] that the *r*^2^_pred_ may, sometimes, not truly reflect the predictive capability of a model on a new dataset. Also, the squared regression coefficient *r*^2^_ts_ between the observed and predicted values of the test set compounds does not necessarily mean that the predicted values are very near to the observed activities. To better evaluate the external predictive capacity of a model, a modified *r*^2^_ts_ term, *r*^2^_m_, is suggested as follows [20]:

(1) $${\text{r}}_{\text{m}}^{2}={\text{r}}_{\text{ts}}^{2}\times (1-\sqrt{{\text{r}}_{\text{ts}}^{2}-{\text{r}}_{\text{o}}^{2}})$$

In case of good external prediction capacity, predicted values will be very close to the observed ones and thus the *r*^2^_ts_ will be very near to the *r*^2^_o_. In the best case *r*^2^_m_ may be equal to *r*^2^_ts_, whereas in the worst case the *r*^2^_m_ value could be zero. Herein, the built GA-RF model achieves a better *r*^2^_m_ value of 0.83 than RF (0.76), which illustrates that the current model possesses a highly predictive power.

### 2.6. Investigation of Parameter Turning on the GA-RF Model

**Figure 3:**
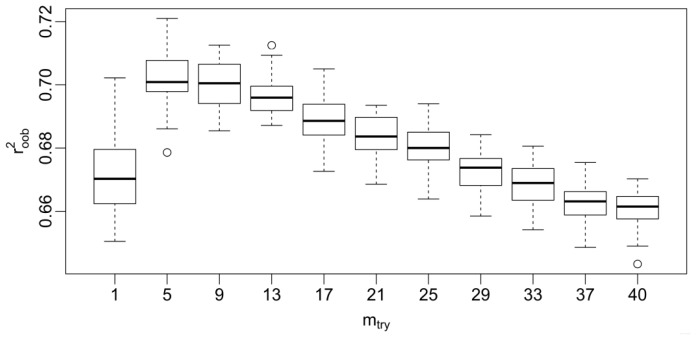
Boxplot of 50 replications of OOB estimation (*r*^2^_oob_) at various values of m_try_. Horizontal lines inside the boxes are the median correlation.

As seen from Tables 2 and 4, since the proposed GA-RF model illustrates relatively better statistical results than the pure RF model, the following analysis is only restrict to this model. Generally, RF has effectively only one tuning parameter, m_try_, which is the number of the descriptors randomly sampled as candidates for splitting at each node during the tree induction. It ranges from 1 to p, the total number of descriptors available, in which p is equal to 40. Although it has been reported [17] that RF still performs well using the default m_try_ value (p/3), one still expects to investigate the effect of parameter turning. Herein, 50 replications of OOB estimation (*r*^2^_oob_) based on the FBPase inhibitors are performed, with the purpose to assess the correlation between the actual and predicted data with a range of m_try_ values, including the default value, which is equal to 13 for the current study. Figure 3 shows the boxplot of these correlations. This plot suggests that m_try_ is optimal when near five with a median value of 0.701, while the default m_tr_*_y_* gives a median value of 0.696. Both results are comparable. It is also observed that the worst statistical results are derived from m_try_ = 1 and = 40. The observation is in agreement with the previous report [17]. From this Figure, one can notice that it is necessary to perform a moderate parameter tuning to get the optimal one, although at most times, RF can give the optimal model by using default parameters.

**Figure 4:**
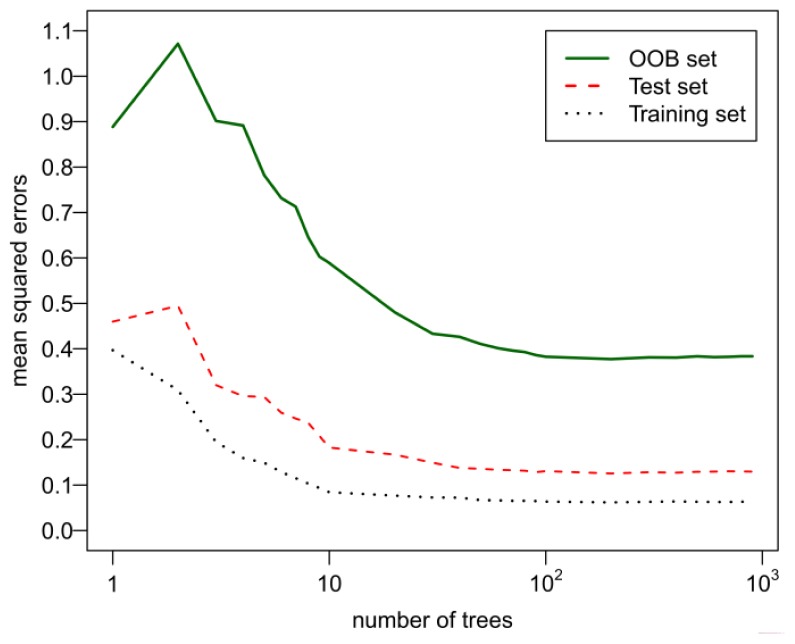
Comparison of mean squared errors from out-of-bag (OOB) set, test set and training set as the number of trees increases for FBPase inhibitors.

Besides the m_try_, the number of trees also has an effect on the RF performance [17]. One principle of building RF model is to ensure that there are sufficient trees in the forest in order to get enough training of each sample. To illustrate this, the performances of OOB set, test set and training set are compared with the increase of the number of trees. Figure 4 shows that similar tendency exists for the tracks of the OOB mean squared errors, the test set and the training set ones, once there are a sufficient number of trees. In the present work, 100 trees are enough to build RF model. The information obtained from this Figure is that mean squared errors of the test and OOB do not increase after the mean squared errors of training set reach the minimum; instead, they converge to their asymptotic values which are also close to their minimum. In this sense, it can be concluded that RF does not overfit, which has been supported by the previous reports [17,23].

### 2.7. Y-Randomization Check

Presently, the Y-randomization check [34] is implemented for further assurance of the robustness of the optimal GA-RF model. The dependent variable is randomly shuffled and a new QSAR model is developed using the original independent variable matrix. The new QSAR models (after several repetitions) are expected to possess low *r*^2^_ncv_, *r*^2^_cv_, *r*^2^_ts_, *r*^2^_pred_, *r*^2^_m_ and high RMSE for the training and test sets, respectively. If the opposite happens, then an acceptable QSAR model cannot be obtained for the specific modeling method and data. In the current work, the Y-randomization check is repeated 500 times and the resulting statistics are compared with the prediction statistics without such checks, with the average values reported in Table 5. As shown in this Table, the correlation coefficients have a significant decline while the RMSE values sharply increase, which indicates that the proposed GA-RF model possesses a real prediction power, and the result is not due to a chance correlation.

### 2.8. Explanation of the Selected Descriptors

**Figure 5:**
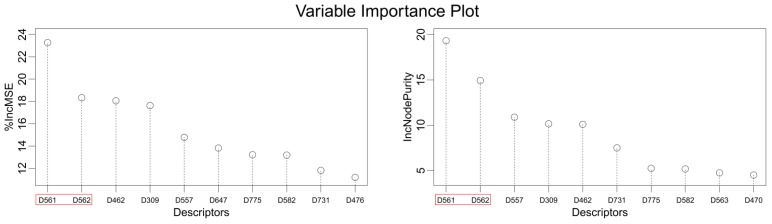
Variable importance plot from GA-RF. The first two important descriptors are surrounded by red frames.

The ideal QSAR model would be robust, sparse, predictive, and interpretable. In many cases such an ideal is not achievable with current descriptors and the corresponding variable mapping methods, although much effort is being expended in approaching this ideal. In most QSAR researches, a full direct explanation for all the descriptors is difficult, where most similar reported works all give few detailed analyses of the descriptors involved in their model development, thus only a few descriptors in this work are explained. Generally speaking, QSAR can be classified into two categories: Interpretative QSAR and predictive QSAR. For the current work, it belongs to the latter. In spite of this fact, we still attempt to offer some rational explanations for the major descriptors using RF built-in variable importance measure technology. Figure 5 depicts the variable importance plot of the GA-RF model. Herein, there are two parameters that give the definitions of the variable importance measures: (1) Mean Decrease Accuracy (%IncMSE) and Mean Decrease Gini (IncNodePurity). The higher values of these two parameters represent the higher variable importance. For more details about these parameters, please refer to the corresponding literature [36,37]. In Figure 5, it can be seen that the first two most important descriptors are D561 and D562 surrounded by the red frames. D561 refers to the lowest eigenvalue from Burden matrix weighted by polarizabilities order-6, while D562 stands for the lowest eigenvalue from Burden matrix weighted by polarizabilities order-7. Both descriptors illustrate that molecular polarizabilities play a central role in FBPase inhibitory activity, which can be supported by the experimental results [29–33]. Previous literature has illustrated that the phosphate group forms a constellation of hydrogen bond interactions that are essential for binding affinity [32]. In the present work, it can be observed that all the studied compounds possess the phosphate group and most of them have oxazole and thiazole groups as well as -NH_2_ substituents, which increase the molecular polarity. In addition, previous reports have also depicted the similar conclusion that the polar groups are favored to increase the FBPase inhibitory activity [16,38]. As suggested in the literature that the FBPase binding pocket presents the hydrophilic nature [11] and the polar groups of inhibitors will bind to the site, leading to potent inhibition of the enzyme. The information obtained by the current work, to some degree, provides an insight into the structural features of both oxazole and thiazole FBPase inhibitors from a theoretical point of view, which should be helpful to design new FBPase inhibitors of this series for the treatment of type 2 diabetes. It should be pointed out that herein we just present a representative explanation of the selected descriptors. However, in terms of developing a highly predictive model, the proposed GA-RF model in this work could implement this task.

## 3. Experimental Section

### 3.1. Dataset

A large, diverse dataset of 190 FBPase inhibitors were collected from the literature [29–33] published by Dang and co-workers after removing duplicated and undesirable compounds. Here the converted molar pIC_50_ (−logIC_50_) values, ranging from 3.60 to 8.00 M, were used as the dependent variables in the QSAR regression analysis to improve the normal distribution of the experimental data points. The whole data set was divided into training (126) and test (64 molecules) sets, respectively. All structures and the corresponding activity values of the dataset as well as their belongings to the training and test sets are listed in Table 3.

### 3.2. Descriptor Calculation

A rational design of novel lead drug is getting more and more popular [39]. QSAR, one of the most frequent drug design methods, can build a bridge between molecular descriptors and statistical methods to predict the new compounds. In the present work, all two-dimensional structures of the dataset were built with the ISIS/Draw 2.3 program, and converted SDF format by the Open Babel software package [40]. The final structures were transferred into Mold^2^ [18], a free program available to the public, to calculate molecular descriptors. The Mold^2^ software package can calculate 777 molecular descriptors solely from 2D chemical structures. Hong *et al*. have reported that the models generated using Mold^2^ descriptors were comparable to those obtained using descriptors from the commercial software packages. In our work, all original 777 molecular descriptors were calculated.

### 3.3. Computational Methods

GA: Genetic algorithm is derived from Darwin’s theory of natural selection and evolution. Based on the Darwinian principle of survival of the fittest, GA obtains the optimal solution after a series of iterative computations including selection or reproduction, crossover or recombination, and mutation. Due to its highly efficient optimization algorithm, GA has already been successfully applied in many QSAR analyses [41–44] to perform variable selection. In the present work, the binary coding form of each chromosome was adopted with 1 and 0 representing selected and non-selected descriptors, respectively. The double point crossover was employed in our study [45–47], which allowed new solution regions in the search space to be explored in order to get the global optimum. In binary code genes, the code may be changed from 0 to 1 or vice versa through mutation operation. As a last step, the old population was replaced by children, and a new generation was produced. The evolutionary process operated for many generations until the termination condition was satisfied [47]. For the detailed methodology about GA, please refer to the corresponding literature [48,49].

RF: Random forest is an ensemble of single decision trees, producing a corresponding number of outputs, and the outputs of all trees are aggregated to obtain one final prediction. The training algorithm of RF for regression can be briefly summarized as follows [17,25]: (1) draw N bootstrap samples from the original training set; (2) construct an un-pruned tree T_p_ (p = 1, …, N) with each training set B_p_. At each node, rather than choosing the best split among all predictors, randomly sample m_try_ of the predictors and then choose the best split from among those variables. The tree is grown to the maximum size and not pruned back; (3) predict the N trees by average for regression. The tree growing algorithm used in RF is CART which is efficient especially when the number of descriptors is very large, with the reason being that RF only tests the m_try_ of the descriptors rather than the whole one, where the default m_try_ is one-third of the number of descriptors for regression. Since the number of m_try_ is very small, the search can finish quickly.

RF possesses its own reliable statistical characteristics based on OOB set prediction, which could be used for validation and model selection with no cross-validation performed. It was shown that the prediction accuracy of an OOB set and a five-fold cross validation procedure was nearly the same [17]. Although RF performs relatively well “off the shelf” without expending much effort on the parameter tuning or variable selection [17], it is also of importance for carrying out some tentative investigations on the changes of m_try_ or descriptor selection to optimize the performance of RF. Herein, we just present a brief introduction of RF, for more details please see the corresponding literature [17,50]. It has been reported that RF can show excellent performance even when most predictive variables are noise, and it can be used when the number of variables is much larger than the number of observations, and it returns measures of variable importance [17,50]. However, to obtain an ideal regression model, a variable selection process is still required. To achieve the above objective, in this work, the GA variable selection method using OOB MSE as the fitness function was carried out to achieve the regression task for the current FBPase inhibitors in order to yield a high prediction model.

### 3.4. Statistical Validation

In the current study, the selected descriptors served as independent variables and the pIC_50_ values as dependent variables in the RF regression analysis. The inner predictive values of the models were evaluated first by a cross-validation process [51,52]. The cross-validated coefficient, *r*^2^_cv_, was calculated using Equation (2):

(2) $${\text{r}}_{\text{cv}}^{2}=1-\frac{\sum_{\text{i}=1}^{\text{train}}{\left({\text{y}}_{\text{i}}-{\hat{\text{y}}}_{\text{i}}\right)}^{2}}{\sum_{\text{i}=1}^{\text{train}}{\left({\text{y}}_{\text{i}}-{\bar{\text{y}}}_{\text{tr}}\right)}^{2}}$$

where y_i_, ŷ_i_, and ȳ_tr_ are the observed, predicted, and mean values of the target property, respectively, for the training set. Herein, $\sum_{\text{i}=1}^{\text{train}}{\left({\text{y}}_{\text{i}}-{\hat{\text{y}}}_{\text{i}}\right)}^{2}$ is the predictive residual sum of squares (PRESS). The optimal number of components obtained from the cross-validation was used to derive the final QSAR model. Then, a non-cross-validation analysis was carried out; and the Pearson coefficient (*r*^2^_ncv_) and RMSE were calculated.

(3) $$\text{RMSE}=\sqrt{\frac{\sum_{\text{i}=1}^{\text{n}}{\left({\text{y}}_{\text{i}}-{\hat{\text{y}}}_{\text{i}}\right)}^{2}}{\text{n}}}$$

where n denotes the number of the studied compounds.

It has been reported [19] that although the low value of *r*^2^_cv_ for the training set can exhibit a low predictive ability of a model, the opposite is not necessarily true. That is, a high *r*^2^_cv_ is necessary, but not sufficient, for a model with a high predictive power. Therefore, the external validation must be estimated to establish a reliable and predictive QSAR model. The predictive coefficient *r*^2^_pred_ listed in the following equation was used to check the models. In addition, various criteria suggested by Tropsha and Roy [19,20] were also performed to validate the predictive power of the current built models.

(4) $${\text{r}}_{\text{pred}}^{2}=1-("\text{PRESS}"/\text{SD})$$

where SD is the sum of the squared deviations between the actual activity of the compounds in the test set and the mean activity in the training set, and “PRESS” is the sum of the squared deviations between predicted and observed activity for each compound in the test set.

## 4. Conclusions

In the present work, a GA-RF algorithm is successfully proposed as an efficient chemoinformatic method to predict FBPase inhibitory activity. The GA-RF model went through all rigorous examinations suggested by Tropsha and Roy with *r*^2^_pred_ of 0.90 and *r*^2^_m_ of 0.83, exhibiting its feasibility and reliability to derive a highly predictive model for FBPase inhibitors. In addition, results from a Y-randomization check illustrate that the GA-RF model possesses real prediction power not due to chance correlation. Explanation of the selected descriptors by GA-RF suggests that the polar factors play a central role in the FBPase inhibition. Thus, the proposed model is useful for predictive tasks to screen for new and potent oxazole and thiazole series of FBPase inhibitors in early drug development.

## Figures and Tables

**Table 1:** Molecular descriptors selected from genetic algorithm-random forest coupled method (GA-RF) for the FBPase inhibitors.

Name	Definition	Name	Definition
D004	Number of 05-membered rings	D543	Lowest eigenvalue from Burdex matrix weighted by van der Waals order-4
D016	Number of double bonds	D545	Lowest eigenvalue from Burdex matrix weighted by van der Waals order-6
D152	Mean atomic polarizability scaled on carbon-SP3	D547	Lowest eigenvalue from Burdex matrix weighted by van der Waals order-8
D164	Index of terminal vertex matrix	D557	Lowest eigenvalue from Burden matrix weighted by polarizabilities order-2
D237	Kier 3-path index	D561	Lowest eigenvalue from Burden matrix weighted by polarizabilities order-6
D279	Total information content order-4 index	D562	Lowest eigenvalue from Burden matrix weighted by polarizabilities order-7
D309	Sum eigenvalue weighted by mass distance matrix	D563	Lowest eigenvalue from Burden matrix weighted by polarizabilities order-8
D455	Geary topological structure autocorrelation length-1 weighted by atomic van der Waals volumes	D571	Highest eigenvalue from Burden matrix weighted by masses order-8
D458	Geary topological structure autocorrelation length-4 weighted by atomic van der Waals volumes	D582	Highest eigenvalue from Burden matrix weighted by electronegativities Sanderson-scale order-3
D462	Geary topological structure autocorrelation length-8 weighted by atomic van der Waals volumes	D589	Highest eigenvalue from Burden matrix weighted by polarizabilities order-2
D465	Geary topological structure autocorrelation length-3 weighted by atomic Sanderson electronegativities	D598	Number of total tertiary carbon-SP3
D470	Geary topological structure autocorrelation length-8 weighted by atomic Sanderson electronegativities	D647	Number of group primary amines (aliphatic)
D473	Geary topological structure autocorrelation length-3 weighted by atomic polarizabilities	D715	Number of group CH2R2
D476	Geary topological structure autocorrelation length-6 weighted by atomic polarizabilities	D719	Number of group CH2RX
D491	Moran topological structure autocorrelation length-5 weighted by atomic van der Waals volumes	D729	Number of group =CHR
D492	Moran topological structure autocorrelation length-6 weighted by atomic van der Waals volumes	D731	Number of group =CHX
D499	Moran topological structure autocorrelation length-5 weighted by atomic Sanderson electronegativities	D746	Number of group H attached to C0(sp3) no X attached to next C
D506	Moran topological structure autocorrelation length-4 weighted by atomic polarizabilities	D754	Number of group O=
D523	Mean molecular topological order-3 charge index	D756	Number of group Al-O-Ar or Ar-O-Ar or R-O-C=X
D541	Lowest eigenvalue from Burden matrix weighted by van der Waals order-2	D775	Hydrophilic factor index

**Table 2:** Statistical performances of GA-RF and RF models a.

Model	Training Set			Test Set		
	
	*r*^2^_ncv_	*r*^2^_cv_	RMSE	*r*^2^_ts_	*r*^2^_pred_	RMSE
GA-RF	0.96	0.67	0.25	0.91	0.90	0.34
RF	0.96	0.59	0.28	0.87	0.85	0.42

a *r*^2^_cv_ from OOB estimation; m_try_ is equal to 13 and 36 for GA-RF and RF, respectively.

**Table 3:** Compounds with their chemical names, observed and predicted activities by GA-RF and RF for the FBPase inhibitors.

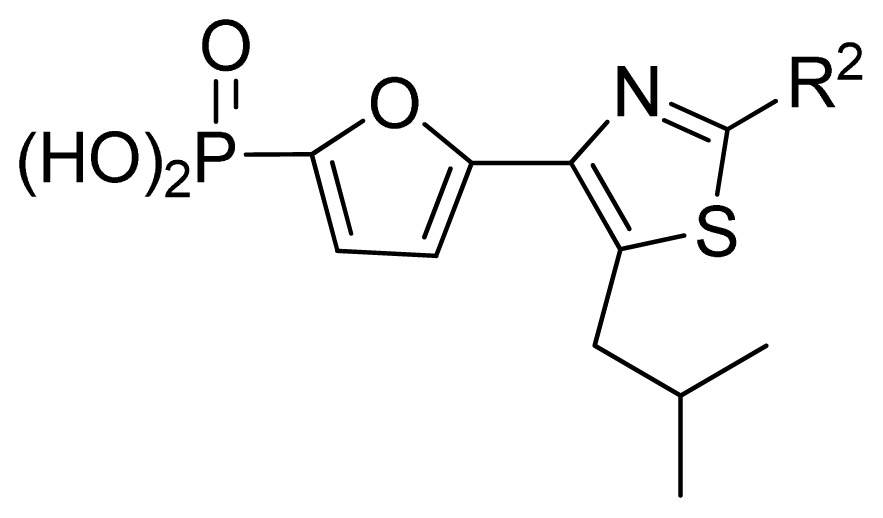										

No.	R^2^		Obs. pIC_50_		GA-RF		RF		Ref. a	
1	Me		7.00		6.62		6.70		[29]	
2 *	Et		6.40		6.20		6.25		[29]	
3	vinyl		5.92		5.99		6.05		[29]	
4	CH_2_OH		6.66		6.63		6.61		[29]	
5 *	H		6.30		6.05		6.27		[29]	
6	Cl		6.74		6.61		6.64		[29]	
7	Br		7.10		6.85		6.89		[29]	
8	SMe		6.05		6.23		6.16		[29]	
9	CN		5.70		5.65		5.73		[29]	
10 *	NH_2_		7.60		7.38		7.20		[29]	
11	NHMe		6.00		5.95		6.08		[29]	
12	NHAc		5.00		5.69		5.66		[29]	
13	CONH_2_		5.56		5.75		6.03		[29]	
14 *	CSNH_2_		6.30		6.38		6.39		[29]	
15	Ph		4.87		5.33		5.40		[29]	
16 *	2-thienyl		5.10		5.93		5.78		[29]	
17	3-pyridyl		5.30		5.40		5.55		[29]	

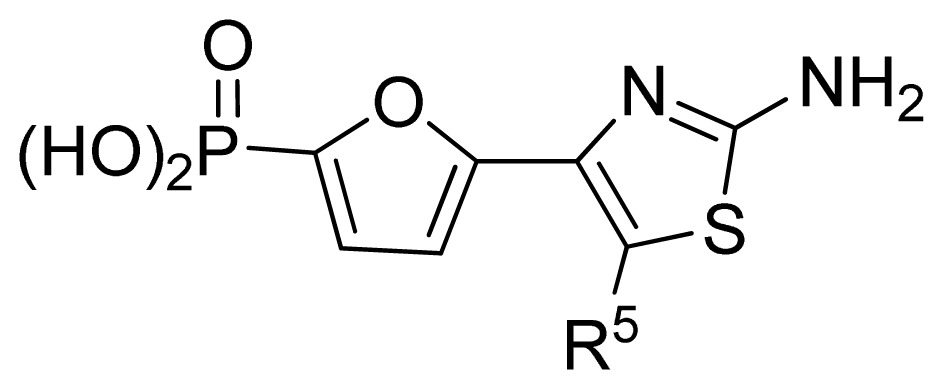										

**No.**	**R^5^**		**Obs. pIC_50_**		**GA-RF**		**RF**		**Ref.** a	

18	H		6.35		6.38		6.42		[29]	
19	Me		6.92		6.84		6.80		[29]	
20	HOCH_2_		6.30		6.73		6.55		[29]	
21 *	*n*-Pr		7.52		7.29		7.13		[29]	
22 *	*i*-Pr		7.55		7.04		7.01		[29]	
23	CF_3_CH_2_		7.24		6.99		7.14		[29]	
24	neopentyl		7.92		7.58		7.51		[29]	
25	cyclobutyl		7.72		7.61		7.54		[29]	
26 *	cyclopentyl		7.68		7.67		7.58		[29]	
27	cyclohexyl		8.00		7.80		7.83		[29]	
28	cyclopropyl-CH_2_		7.70		7.62		7.53		[29]	
29	cyclopentyl-CH_2_		7.74		7.36		7.44		[29]	
30	cyclohexyl-CH_2_		7.23		7.18		7.08		[29]	
31	PhCH_2_		6.82		6.85		6.82		[29]	
32 *	morpholinyl-CH_2_		6.25		6.16		6.45		[29]	

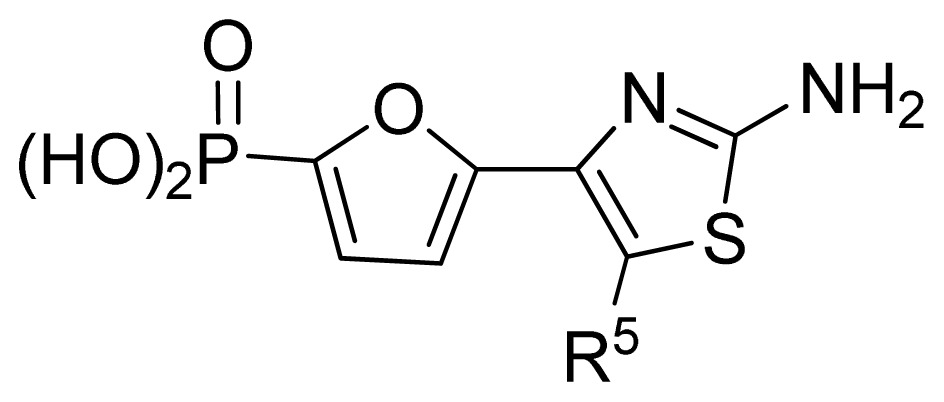										

**No.**	**R^5^**		**Obs. pIC_50_**		**GA-RF**		**RF**		**Ref.**^a^	

33	Cl		7.15		7.03		6.97		[29]	
34 *	Br		7.30		6.99		6.88		[29]	
35 *	I		7.00		6.87		6.36		[29]	
36	1-morpholinyl		7.80		7.09		7.29		[29]	
37	EtS		7.48		7.32		7.24		[29]	
38 *	*n*-PrS		7.80		7.21		7.03		[29]	
39	*i*-PrS		7.62		7.50		7.46		[29]	
40	*t*-BuS		7.62		7.52		7.53		[29]	
41 *	PhS		6.52		6.70		6.58		[29]	
42	CONMe_2_		5.77		5.94		6.22		[29]	
43	CO_2_Et		7.85		7.55		7.48		[29]	
44	CO_2_Bn		7.82		7.25		7.43		[29]	
45	*n*-PrSO		6.07		6.56		6.45		[29]	
46 *	Ph		7.85		7.68		7.64		[29]	
47 *	2-MeO-Ph		7.37		7.51		7.52		[29]	
48	3-MeO-Ph		7.68		7.60		7.62		[29]	
49	4-MeO-Ph		7.66		7.61		7.64		[29]	
50 *	4-MeS-Ph		7.68		7.41		7.40		[29]	
51	4-*t*-Bu-Ph		7.06		7.21		7.10		[29]	
52 *	4-MeO_2_C-Ph		7.85		7.48		7.36		[29]	
53	4-F-Ph		7.80		7.71		7.68		[29]	
54	4-Cl-Ph		7.89		7.76		7.75		[29]	
55	4-Ac-Ph		7.49		7.45		7.48		[29]	
56	4-MeSO_2_-Ph		7.39		7.30		7.00		[29]	
57 *	4-Ph-Ph		7.47		7.31		7.23		[29]	
58	2-nathphyl		7.92		7.66		7.61		[29]	
59	2-furanyl		7.40		7.12		7.22		[29]	
60 *	2-thienyl		7.36		7.17		7.20		[29]	

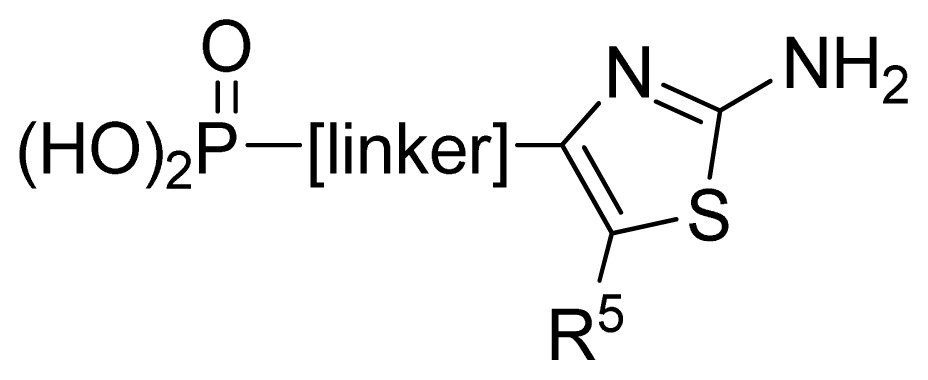										

**No.**	**[linker]**		**R^5^**		**Obs. pIC_50_**		**GA-RF**	**RF**	**Ref.** a	

61	2,5-furanyl		H		5.00		5.41	5.78	[29]	
62	-CH_2_OCO-		*n*-Pr		7.30		6.90	6.92	[29]	
63*	-CH_2_NHCO-		2-thienyl		6.02		6.42	6.69	[29]	
64	2,6-pyridyl		H		5.70		5.74	5.94	[29]	
65	1,3-phenyl		H		5.89		6.06	6.01	[29]	
66 *	1,3-phenyl-(6-Me)		*n*-Pr		6.87		6.71	6.39	[29]	
67 *	1,3-phenyl-(6-OMe)		*i*-Pr		6.68		7.05	6.89	[29]	
68 *	1,3-phenyl-(6-F)		Ph		7.10		7.42	7.27	[29]	

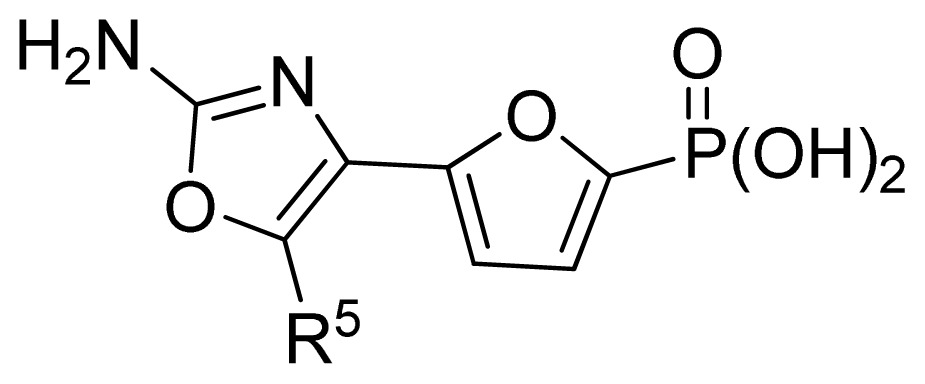										

**No.**	**R^5^**		**Obs. pIC_50_**		**GA-RF**		**RF**		**Ref.** a	

69 *	*i*-Bu		6.92		6.38		6.13		[30]	
70	H		5.00		5.60		5.43		[30]	
71	Allyl		6.85		6.70		6.51		[30]	
72	*n*-Bu		6.77		6.64		6.54		[30]	
73*	*n*-Pentyl		6.68		6.54		6.35		[30]	
74	-CH_2_-cyclohexyl		6.49		6.24		6.29		[30]	
75	Ph		6.80		6.78		6.80		[30]	
76	Bn		6.05		6.23		6.12		[30]	
77	-CH_2_-(2-thienyl)		6.59		6.47		6.59		[30]	
78	*n*-PrS		7.15		6.97		6.91		[30]	
79	*i*-PrS		6.96		7.01		7.02		[30]	
80 *	*t*-BuS		6.92		6.64		7.05		[30]	
81	PhS		5.40		5.79		6.08		[30]	
82	-CO_2_Me		7.17		7.06		6.70		[30]	
83 *	-CO_2_Et		7.42		7.10		6.85		[30]	
84	-CO_2_Pr-*i*		7.40		7.13		7.14		[30]	
85	-CO_2_Bn		7.07		6.95		6.91		[30]	
86	-COSEt		7.52		7.23		7.20		[30]	
87	-COBu-*t*		6.07		6.10		6.22		[30]	

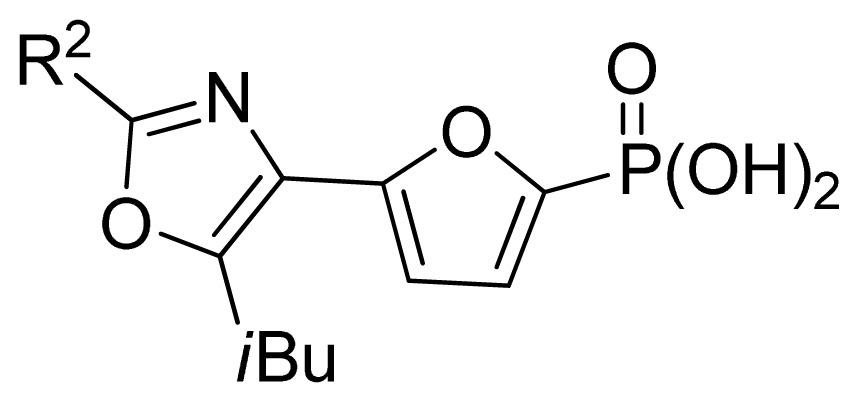										

**No.**	**R^2^**		**Obs. pIC_50_**		**GA-RF**		**RF**		**Ref.** a	

88	Me		6.22		6.24		6.14		[30]	
89	HO		5.00		5.48		5.43		[30]	
90 *	H		5.72		5.87		5.80		[30]	
91	Me_2_N-		5.68		5.61		5.54		[30]	
92*	*i*-Pr-		5.66		5.79		5.78		[30]	
93	MeHN-		5.37		5.55		5.62		[30]	
94	Et		6.02		6.09		5.94		[30]	
95 *	EtHN-		5.00		5.43		5.68		[30]	
96	vinyl		5.17		5.49		5.54		[30]	

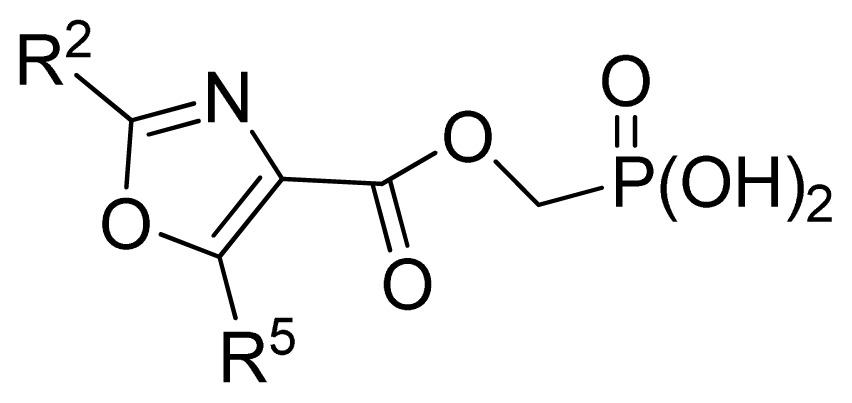										

**No.**	**R^2^**		**R^5^**	**Obs. pIC_50_**		**GA-RF**	**RF**		**Ref.** a	

97	H_2_N-		H	5.15		5.50	5.43		[30]	
98 *	H_2_N-		Me	6.38		6.19	5.72		[30]	
99	H_2_N-		Et	6.42		6.39	6.17		[30]	
100 *	H_2_N-		*n*-Pr	6.55		6.46	6.08		[30]	
101 *	H_2_N-		*i*-Pr	6.24		6.36	6.19		[30]	
102 *	H_2_N-		*n*-Bu	6.60		6.32	5.98		[30]	
103 *	H_2_N-		*n*-Pent	6.46		6.30	6.10		[30]	
104	Me		CF_3_	5.00		5.45	5.54		[30]	
105	H		Ph	5.00		5.35	5.45		[30]	

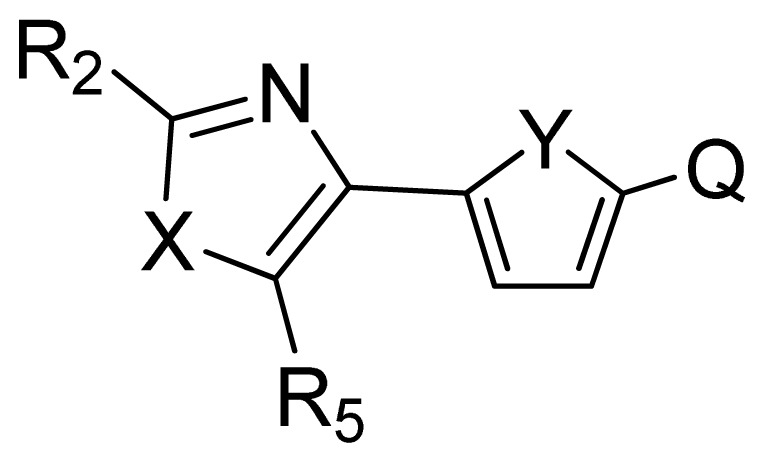										

**No.**	**X**	**Y**	**Q**	**R^2^**	**R^5^**	**Obs. pIC_50_**	**GA-RF**	**RF**		**Ref.** a

106	NH	O	PO_3_H_2_	NH_2_	*i*Bu	5.30	5.55	5.47		[31]
107	S	O	PO_3_H_2_	H	H	5.26	5.58	5.56		[31]
108	CH=CH	O	PO_3_H_2_	NH_2_	Ph	7.38	6.95	6.87		[31]

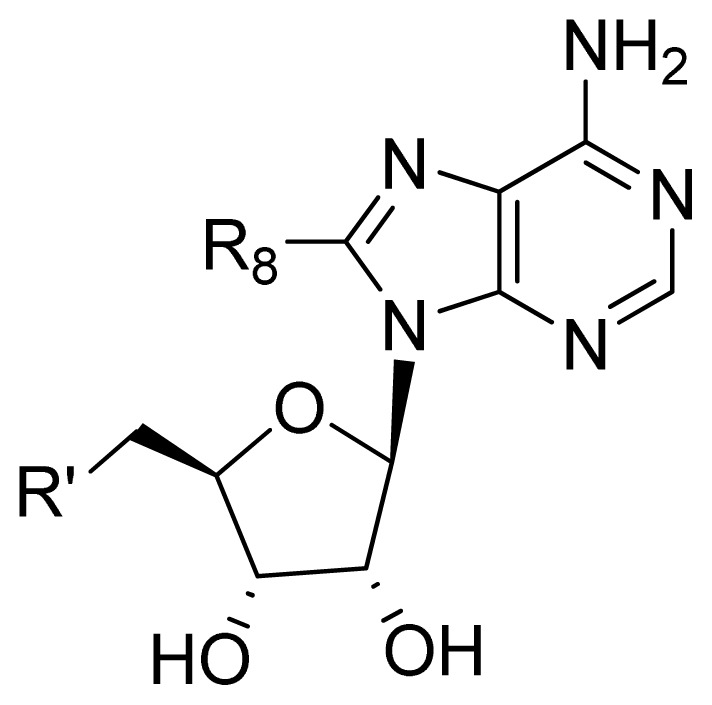										

**No.**	**R^8^**		**R^′^**	**Obs. pIC_50_**	**GA-RF**		**RF**			**Ref.** a

109 *	-NH(CH_2_)_2_PO_3_H_2_		OH	4.00	4.36		4.62			[32]
110 *	-NH(CH_2_)_2_OPO_3_H_2_		OH	3.85	4.27		4.56			[32]
111	-NH(CH_2_)_2_PO_3_H_2_		H	4.00	4.27		4.44			[32]

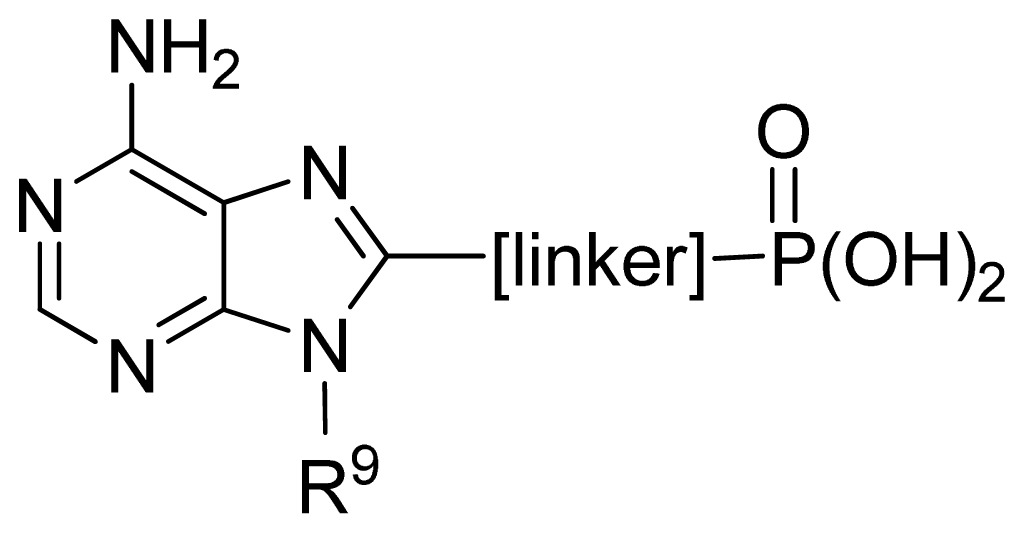										

**No.**	**[linker]**		**R^9^**	**Obs. pIC_50_**		**GA-RF**		**RF**		**Ref.** a

112	-NH(CH_2_)_2_-		Bn	4.04		4.20		4.26		[32]
113 *	-NH(CH_2_)_2_-		Ph(CH_2_)_2_-	4.00		4.14		4.20		[32]
114	-NH(CH_2_)_2_-		2-naphthyl-CH_2_-	4.46		4.35		4.42		[32]
115 *	-CONHCH_2_-		Ph(CH_2_)_2_-	4.00		4.19		4.50		[32]
116	-(CH_2_)_3_-		Ph(CH_2_)_2_-	4.00		4.04		4.16		[32]
117 *	-CH=CHCH_2_-		Ph(CH_2_)_2_-	4.00		4.19		4.28		[32]
118	-S(CH_2_)_2_-		Ph(CH_2_)_2_-	3.84		4.03		4.29		[32]
119 *	-CH_2_OCH_2_-		Ph(CH_2_)_2_-	4.64		4.09		4.38		[32]
120	-2,5-furanyl-		Ph(CH_2_)_2_-	5.30		4.95		4.95		[32]
121	-2,5-thienyl-		Ph(CH_2_)_2_-	4.32		4.55		4.71		[32]

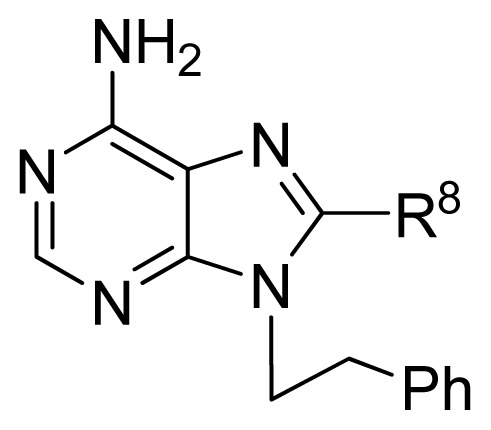										

**No.**		**R^8^**		**Obs. pIC_50_**	**GA-RF**		**RF**	**Ref.** a		

122 *		-(CH_2_)_2_-OPO(OH)_2_		4.40	4.13		4.21	[32]		
123		-2,5-furanyl-SO_3_H		3.82	4.43		4.50	[32]		

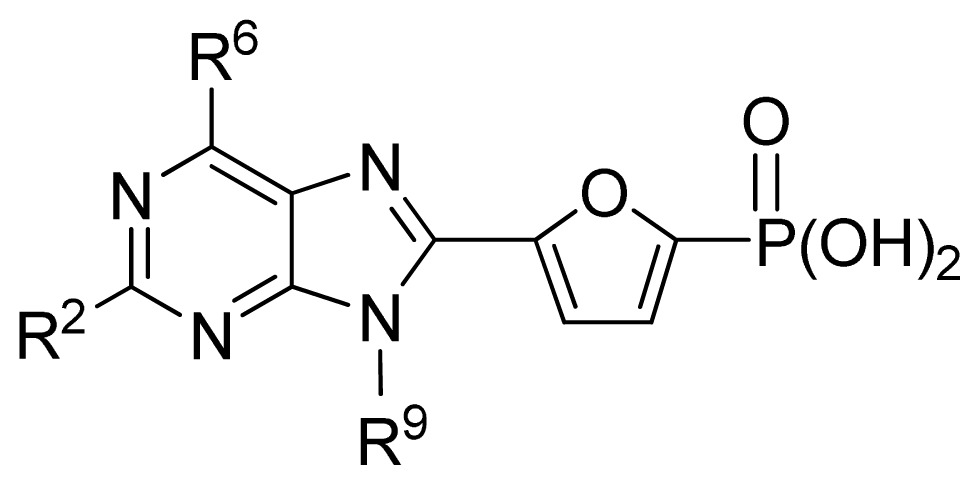										

**No.**	**R^2^**	**R**		**R^9^**	**Obs. pIC_50_**		**GA-RF**	**RF**		**Ref.** a

124	H	-N(Me)_2_		-(CH_2_)_2_Ph	3.60		4.11	4.17		[32]
125	H	-NHMe		-(CH_2_)_2_Ph	4.30		4.46	4.41		[32]
126	H	Cl		-(CH_2_)_2_Ph	4.30		4.61	4.59		[32]
127	H	-NH_2_		-CH_2_CH(Ph)_2_	4.15		4.31	4.47		[32]
128	H	-NH_2_		-(CH_2_)_2_(cyclohexyl)	5.85		5.54	5.54		[32]
129	H	-NH_2_		-(CH_2_)(2-naphthyl)	5.48		5.22	5.19		[32]
130	H	-NH_2_		cyclopropyl	5.82		5.70	5.78		[32]
131	H	-NH_2_		cyclopentyl	5.70		5.69	5.76		[32]
132	H	-NH_2_		Et	5.74		5.65	5.76		[32]
133	H	-NH_2_		isobutyl	5.82		5.81	5.82		[32]
134	H	-NH_2_		neopentyl	6.10		5.87	5.87		[32]
135 *	-SMe	-NH_2_		isobutyl	6.15		5.52	5.42		[32]
136	-SO_2_Me	-NH_2_		isobutyl	4.55		4.95	4.99		[32]

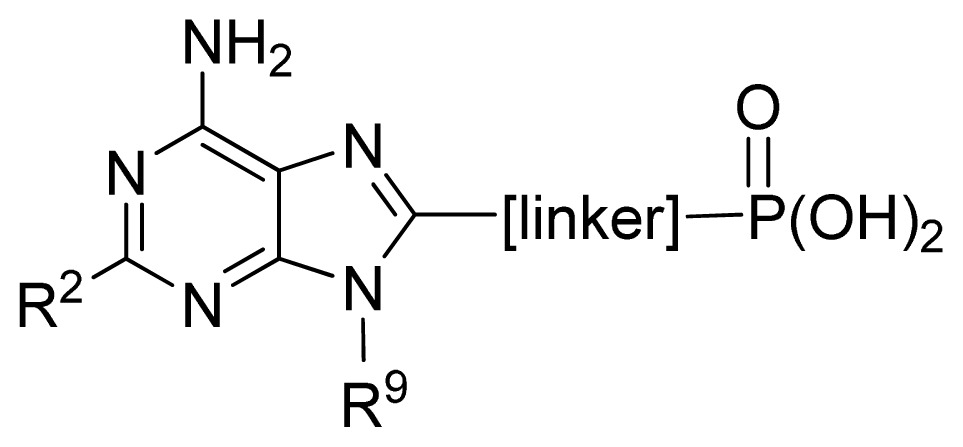										

**No.**	**R^2^**	**R^9^**		**[linker]**		**Obs. pIC_50_**		**GA-RF**	**RF**	**Ref.** a

137 *	H	-CH_2_C(Me)_2_CH_2_OH		2,5-furanyl		5.35		5.52	5.40	[32]
138	H	-CH_2_C(Me)_2_CH_2_Cl		2,5-furanyl		6.05		5.83	5.91	[32]
139 *	H	-CH_2_C(Me)_2_CMe_3_		2,5-furanyl		5.80		5.66	5.67	[32]
140 *	H	-CH(Me)CMe_3_		2,5-furanyl		5.30		5.72	5.74	[32]
141	-NH_2_	-CH_2_CMe_3_		2,5-furanyl		5.26		5.27	5.27	[32]
142 *	-SMe	-CH_2_CMe_3_		2,5-furanyl		5.96		5.54	5.42	[32]
143 *	H	-CH_2_CMe_3_		2,5-(3,4-di-Cl)furanyl		4.89		5.56	5.42	[32]

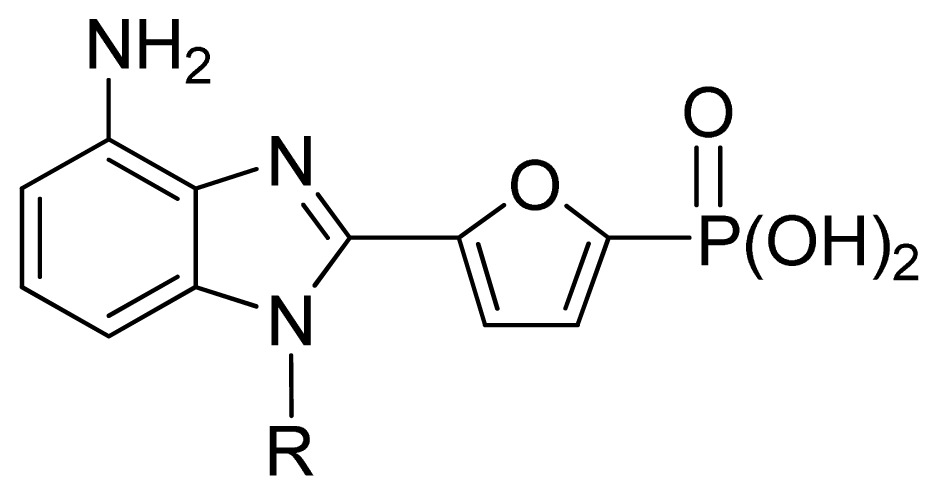										

**No.**		**R**		**Obs. pIC_50_**		**GA-RF**		**RF**	**Ref.** a	

144 *		Me		5.22		5.49		5.70	[33]	
145		Et		5.65		5.80		5.88	[33]	
146		*n*Pr		5.96		6.00		6.03	[33]	
147 *		*i*Bu		5.82		5.91		6.00	[33]	
148		cycllopropyl-CH_2_-		6.10		6.03		6.02	[33]	
149		cyclobutyl-CH_2_-		6.10		6.04		6.01	[33]	
150		cyclopentyl-CH_2_-		5.82		5.87		5.81	[33]	
151		cyclohexyl-CH_2_-		5.60		5.64		5.63	[33]	
152		cycloheptyl-CH_2_-		5.49		5.57		5.64	[33]	
153		norbornyl		6.00		5.94		5.85	[33]	
154 *		benzyl		5.30		5.72		5.70	[33]	
155		4-*t*Bu-benzyl		5.02		5.26		5.34	[33]	
156		4-CF_3_-benzyl		5.15		5.50		5.51	[33]	
157		4-Ph-benzyl		5.60		5.63		5.59	[33]	
158 *		3-furanyl-CH_2_-		5.38		5.74		5.68	[33]	
159 *		3-HO-benzyl		5.73		5.87		5.75	[33]	
160 *		3-thienyl-CH_2_-		5.40		6.02		6.08	[33]	

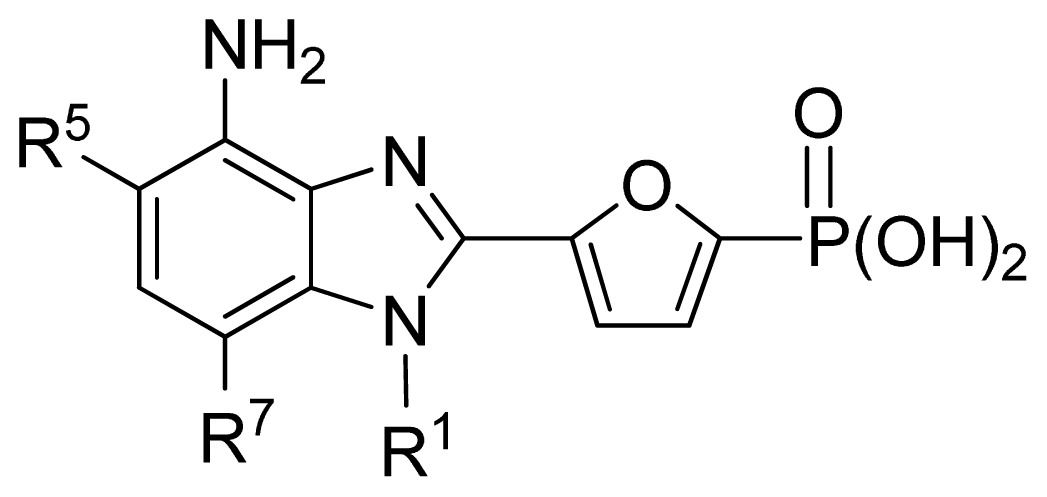										

**No.**	**R^1^**	**R^5^**	**R^7^**	**Obs. pIC_50_**		**GA-RF**		**RF**		**Ref.** a

161 *	*i*Bu	Et	H	5.60		5.86		5.87		[33]
162	*i*Bu	*n*Pr	H	5.52		5.69		5.66		[33]
163 *	*i*Bu	MeO	H	6.15		6.41		6.25		[33]
164	*i*Bu	OH	H	6.30		6.23		6.24		[33]
165	*i*Bu	Cl	H	6.70		6.52		6.56		[33]
166	*i*Bu	H	Cl	6.05		6.19		6.11		[33]
167	*i*Bu	Br	H	6.40		6.32		6.29		[33]
168 *	*i*Bu	H	Br	6.40		6.21		6.14		[33]
169 *	*i*Bu	F	H	7.00		6.56		6.47		[33]
170 *	(Et)_2_CHCH_2_-	F	H	6.82		6.83		6.57		[33]
171	(Et)_2_CH-	F	H	6.07		6.42		6.38		[33]
172 *	*c*Pr-CH_2_-	F	H	7.26		6.54		6.53		[33]

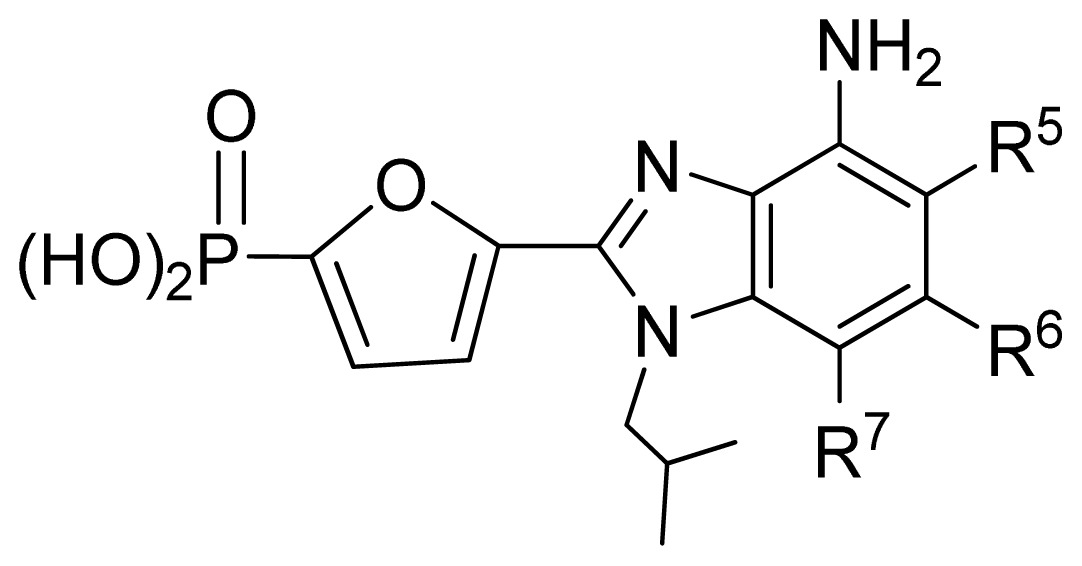										

**No.**	**R^5^**	**R^6^**	**R^7^**	**Obs. pIC_50_**		**GA-RF**		**RF**		**Ref.** a

173	Br	H	Br	6.00		6.09		6.03		[33]
174	Cl	H	Cl	6.35		6.34		6.30		[33]
175 *	F	H	Cl	7.00		6.77		6.67		[33]
176	F	H	Br	6.89		6.75		6.67		[33]
177	F	Cl	H	6.65		6.66		6.59		[33]
178	Br	Cl	Cl	5.00		5.53		5.60		[33]
179 *	F	H	vinyl	6.55		6.90		6.94		[33]
180	F	H	*c*Pr	7.22		7.08		7.10		[33]

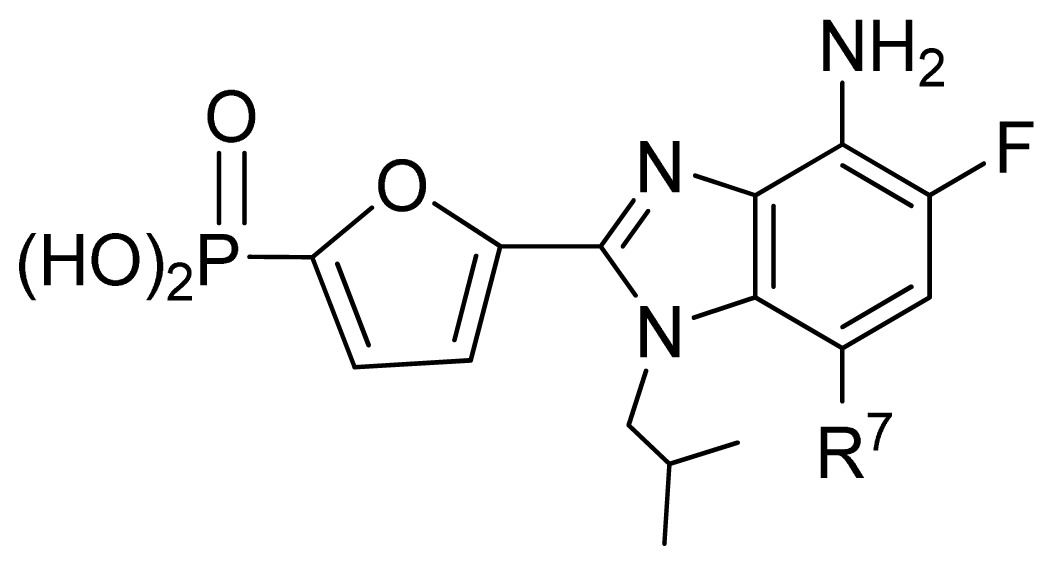										

**No.**	**R^7^**		**Obs. pIC_50_**		**GA-RF**		**RF**		**Ref.** a	

181 *	Ph		7.05		6.79		6.83		[33]	
182	4-F-Ph		6.74		6.74		6.75		[33]	
183	4-Cl-Ph		7.05		6.94		6.81		[33]	
184	Et		7.26		7.07		7.06		[33]	
185	*n*Pr		7.00		6.99		7.02		[33]	
186	*t*Bu(CH_2_)_2_-		6.68		6.70		6.67		[33]	
187	(Me)_2_CH(CH_2_)_3_-		7.00		6.92		6.99		[33]	
188	HO(CH_2_)_3_-		7.10		6.97		7.05		[33]	
189	(Me)_2_N(CH_2_)_3_-		7.26		6.72		6.73		[33]	
190 *	Cl(CH_2_)_4_-		7.15		6.74		6.78		[33]	

* test set;

a from the corresponding references.

**Table 4:** External predictability of GA-RF model.

Model	*r*^2^_ts_	*r*^2^_pred_	*r*^2^_o_	(*r*^2^_ts_ − *r*^2^_o_)/*r*^2^_ts_	k	*r*^2^_m_
GA-RF	0.91	0.90	0.90	0.01	1.01	0.83
RF	0.87	0.85	0.85	0.02	1.01	0.76

**Table 5:** Comparison with and without Y-randomization check of the optimal GA-RF model.

Model	Training Set			Test Set			
	
	*r*^2^_ncv_	*r*^2^_cv_	RMSE	*r*^2^_ts_	*r*^2^_pred_	*r*^2^_m_	RMSE
GA-RF a	0.96	0.67	0.25	0.91	0.90	0.83	0.34
GA-RF b	0.01	−0.14	1.27	0.06	−0.10	0.04	1.13

a without Y-randomization check;

b with Y-randomization check.
